# Acetic Extracts

**Published:** 1843-07-08

**Authors:** 


### ACETIC EXTRACTS.

M. Ferrari assures us that extracts of aconite, cicuta, hyosciamus, and stramonium, prepared with distilled vinegar, are much more active than those made with water. He has obtained still more active extracts by treating the plants with alcohol at 36°, containing 1-36th part of its weight of pyroligneous acid.—*Prov. Med. Journ.*

